# PCR-based genotyping of *Helicobacter pylori *of Gambian children and adults directly from biopsy specimens and bacterial cultures

**DOI:** 10.1186/1757-4749-3-5

**Published:** 2011-04-20

**Authors:** Ousman Secka, Martin Antonio, Mary Tapgun, Douglas E Berg, Christian Bottomley, Vivat Thomas, Robert Walton, Tumani Corrah, Richard A Adegbola, Julian E Thomas

**Affiliations:** 1 Bacterial Diseases Programme, Medical Research Council Laboratories, The Gambia; 2 School of Clinical Medical Sciences, Newcastle University, Newcastle upon Tyne, UK; 3 Department of Molecular Microbiology, Washington University School of Medicine, St. Louis, USA; 4 London School of Hygiene and Tropical Medicine, London, UK; 5 Bill & Melinda Gates Foundation, Seattle, USA

**Keywords:** Genotyping, *Helicobacter pylori*, biopsy specimens, bacterial cultures

## Abstract

**Background:**

*Helicobacter pylori *is an important agent of gastroduodenal disease in Africa and throughout the world. We sought to determine an optimum method for genotyping *H. pylori *strains from children and adults in The Gambia, West Africa.

**Results:**

Virulence genes were amplified in 127 of 190 cases tested (121 adults and 6 children); each of 60 bacterial cultures, and 116 from DNA extracted directly from biopsies. The proportion of biopsies that were *cagA*+, the ratio of *vacAs1*/*s2*, and *vacAm1*/*m2*, and the proportion of mixed strain populations in individual subjects changed with age. Strains lacking virulence *cagA *and *vacA *genes and with apparently homogeneous (one predominant strain) infections were more common among infants than adults.

**Conclusions:**

In order to detect the range of bacterial genotypes harbored by individual patients, direct PCR proved slightly superior to isolation of *H. pylori *by biopsy culture, but the techniques were complementary, and the combination of both culture and direct PCR produced the most complete picture. The seemingly higher virulence of strains from adult than infant infections in The Gambia merits further analysis.

## Background

*Helicobacter pylori *chronically infects over 50% of people worldwide, causes gastritis and sometimes gastric or duodenal ulceration, and increases the risk of gastric cancer [1,2]. Infection also contributes to other maladies such as malnutrition among the very poor, iron deficiency anemia, and susceptibility to other food and water borne pathogens, especially in developing countries, including The Gambia [3,4]. The prevalence of *H. pylori *infection is particularly high in developing countries including The Gambia [5-7]. *H. pylori*, because it is a fastidious micro-aerobic bacterium, it is technically difficult to grow and maintain for molecular biologic research in poorly resourced laboratories in Africa. These challenges coupled with the uniqueness of genotypes of African strains and special features of human physiology and environment in this continent limit our understanding of the spectrum of *H. pylori*-associated diseases and how this is affected by bacterial genotype in Africa [8,9]. So extensive efforts have been made to determine an optimum method for PCR-based genotyping of *H. pylori *[10-13]. To more effectively investigate the influence *of H. pylori *genotype on associated diseases in a West African setting, this study sought to determine an optimum method for PCR-based genotyping of *H. pylori *in The Gambia, West Africa.

## Results

A total of 169 biopsy samples from adult subjects, and 21 from infants were investigated for *H. pylori *infections by both culture and PCR of DNA obtained directly from biopsies. 89/169 (52.6%) adults seemed to be culture positive. Pure *H. pylori *cultures were obtained from only 63 of them, but not the other 26, primarily because of overgrowth by contaminants despite inclusion of multiple antibiotics in the culture medium or bacterial cells failing to survive further subculture. Direct PCR from adult biopsies indicated that 164/169 (97%) were positive for *Hp16s *(table 1). The DNA extracts from the remaining 5 biopsies were *H. pylori *negative with *Hp16s *and did not amplify for any of the genes tested, even though they were culture positive. These five samples were tested for PCR inhibitors by spiking them with DNA from a known positive. The spiked samples were also all negative after PCR (data not shown), which implies the presence of a potent inhibitor, possibly a ribonuclease. Virulence gene data were obtained by direct PCR from both biopsies and cultures in 60 cases and these data are used for the comparisons in table 2.

**Table 1:** Comparison between culture results and direct PCR with *Hp specific 16s *primer

Result	Culture	Direct by PCR
Positive	89 (52.6%)	164 (97.0%)

Negative	80	5

Total	169	169

**Table 2:** Comparison of amplification of virulence genes between PCR on bacterial cultures and direct PCR on biopsy material

PCR on Biopsy						
**Culture**	***cagA*+**	***cag PAI*** ***empty*** ***site***	***cagA*+** ***cag PAI*** ***empty*** ***site***	**No** **amplification** **with primer**	**% Agreement** **(95% CI)**	**Kappa (95% CI)**

*CagA*+	31(52)	0(0)	3(5)	3(5)		

*cag PAI* *empty site*	0(0)	12(20)	0(0)	2(3)		

*cagA*+ *cag* *PAI empty*	4(7)	1(2)	2(3)	2(3)	84.9(75.3,94.5)	0.70(0.50,0.91)

						

	*s1*	*s2*	*s1*&*s2*	Missing		

*s1*	31(52)	4(7)	0(0)	5(8)		

*s2*	5(8)	9(15)	0(0)	1(2)		

*s1*&*s2*	3(5)	1(2)	0(0)	1(2)	75.5(63.9,87.1)	0.45(0.21,0.68)

						

	*m1*	*m2*	*m1*&*m2*	Missing		

*m1*	18(30)	1(2)	1(2)	6(10)		

*m2*	1(2)	17(28)	0(0)	2(3)		

*m1*&*m2*	4(7)	5(8)	3(5)	2(3)	76.0(64.2,87.8)	0.62(0.42,0.82)

						

	*iceA1*	*iceA2*	*icA1*&*2*	Missing		

*iceA1*	5(8)	0(0)	1(2)	3(5)		

*iceA2*	1(2)	11(18)	29(48)	7(12)		

*iceA1*&*2*	0(0)	3(5)	0(0)	0(0)	32.0(19.1,44.9)	0.06(-0.05,0.16)

Amongst the 80 culture negative adult subjects for whom *Hp16s *was positive, amplification of some or all virulence genes was only achieved in 20 cases. It is not yet known if these strains lacked virulence genes, were divergent in primer binding sequences, or the bacterial density was so low that amplification was possible only with the most general of primer combinations, such as *Hp16s*. The remaining 60 samples did not show amplification for any of the genes tested despite a positive response to *Hp16s*.

Amongst 21 infants, 8 were *Hp16s *positive and pure *H. pylori *cultures were obtained successfully from six of them. We succeeded in amplifying virulence gene sequences only from the 6 culture positive children. Direct PCR of the biopsies from the other 13 children were either negative (n = 10) or not done (n = 3).

**Figure 1:**
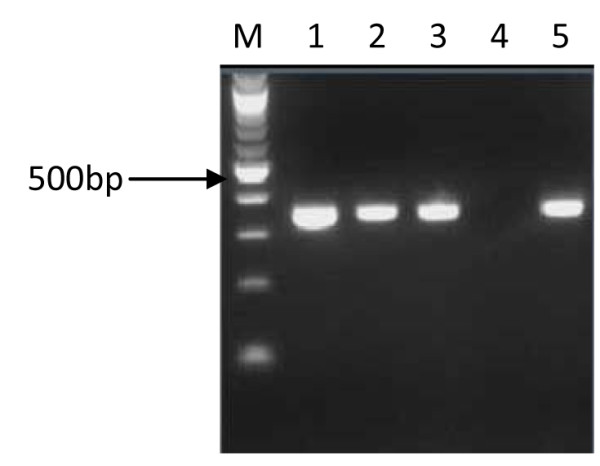
**PCR inferred results of *cagA *gene**. 1.5% gel electrophoresis of *H. pylori *genotypes showing PCR results of *cagA *gene. Lane M is a 100 bp ladder (Biolabs, UK); lanes 1, 2, 3 and 5 showed PCR products (349 bp) of *cagA *genes, lane 4 is *cagA *negative.

**Figure 2:**
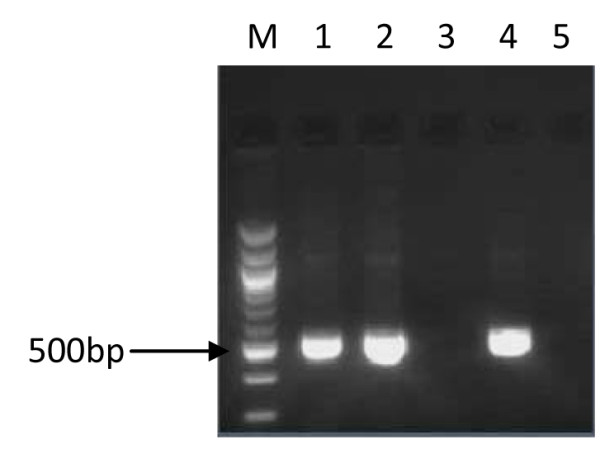
**PCR inferred results of *cag emptysite***. 1.5% gel electrophoresis of *H. pylori *genotypes showing PCR results of *cag emptysite*. Lane M is a 100 bp ladder (Biolabs, UK), lanes 1, 2 and 4 showed PCR products of 535 bp indicating the presence of *cag emptysite*, lanes 3 and 5 were *cag emptysite *negative.

**Figure 3:**
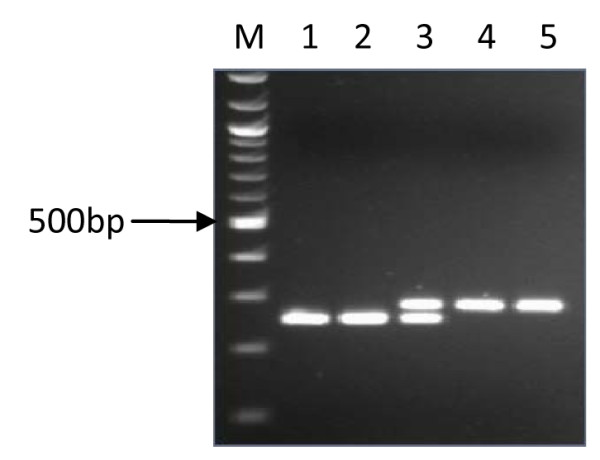
**PCR inferred results of signal region of *vacA *gene**. 1.5% gel electrophoresis of *H. pylori *genotypes showing PCR results of *s1 *and *s2 *allelles of *vacA *gene. Lane M is a 100 bp ladder (Biolabs, UK), lane 1 and 2 showed the presence of *s1 *(259 bp), lane 3 is both *s1 *and *s2 *positive and lane 4 and 5 are *s2 *(289 bp) positive.

**Figure 4:**
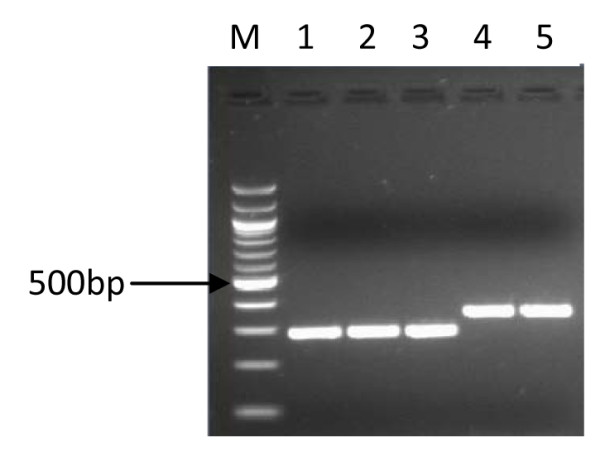
**PCR inferred results of mid region of *vacA *gene**. 1.5% gel electrophoresis of *H. pylori *genotypes showing PCR results of *m1 *and *m2 *allelles of *vacA *gene. Lane M is a 100 bp ladder (Biolabs, UK), lanes 1, 2 and 3 are *m1 *positive; lanes 4 and 5 showed the presence of *m2*.

**Figure 5:**
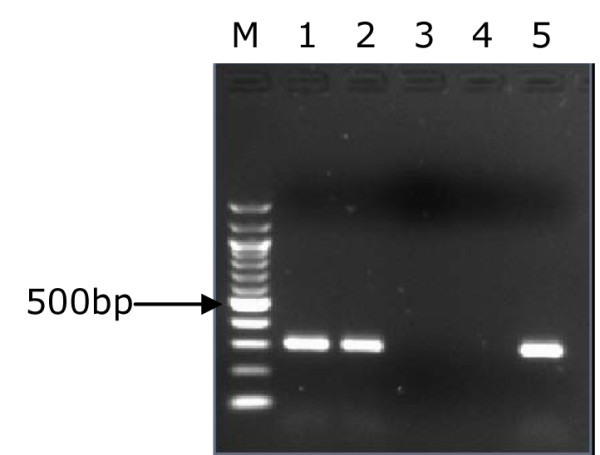
**PCR inferred results *iceA1***. 1.5% gel electrophoresis of *H. pylori *genotypes showing PCR results of *iceA1 *gene. Lane M is a 100 bp ladder (Biolabs, UK), lanes 1, 2 and 5 showed the presence of 297 bp of *iceA1*, lanes 3 and 4 are *iceA1 *negative.

**Figure 6:**
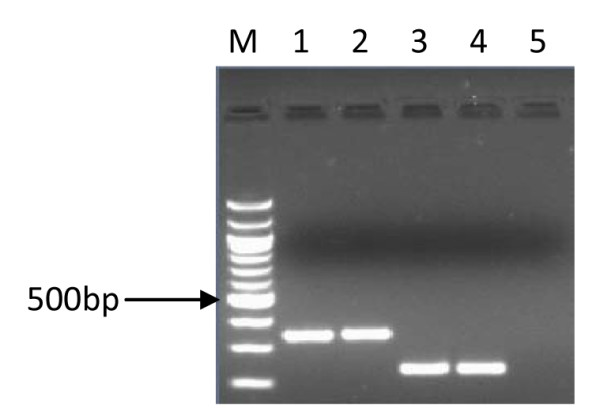
**PCR inferred results *iceA2***. 1.5% gel electrophoresis of *H. pylori *genotypes showing PCR results of *iceA2*. Lane M is a 100 bp ladder (Biolabs, UK), lanes 1 and 2 showed the presence of 334 bp of *iceA2 *and lanes 3 and 4 were *iceA2 *positive of 229 bp, lane 5 was *iceA2 *negative.

Virulence gene amplification was successful in 127 (121 adults and 6 children) cases. A comparison of the products that were indicative of *cagA *(Figure 1), *cag emptysite *(Figure 2), *vacAs *alleles (Figure 3), *vacAm *alleles (Figure 4), *iceA1 *(Figure 5) and *iceA2 *(Figure 6) between both methods for detecting *H. pylori *is summarized for 60 samples for which sufficient amplified DNA was obtained for this further analysis. The proportion of samples that were *cagA*+ve with DNA from biopsies and from culture was similar, 58.3% and 61.7% respectively. The success in amplification of *vacAs1*/*s2 *(*s1 *= toxigenic vs *s2 *= non-toxigenic), *vacAm1*/*m2 *and *iceA1 *alleles was similar from cultures and corresponding biopsies, and agreements between genotypes inferred using DNAs directly from these two sources was good for both *cagA *and *m1, m2 *alleles of *vacA*, moderate for *s1*, *s2 *alleles of *vacA*, and poor for *iceA*. The poor agreement in the *iceA *analysis stemmed from the many classified only as *iceA2 *by PCR from bacterial culture but *iceA1 *and *iceA2 *by biopsy which could have been due to the fact that certain bacterial strains in a mixed infection grew much better than others in culture. In direct PCR up to 16.7% of culture positive biopsies failed to amplify DNA for individual alleles.

The proportion of biopsies that were *cagA*+, the proportion of *vacAs1*, and *vacAm1*, and the proportion of mixed cultures from individual subjects varied with age. Table 3 is a summary of all PCR results, including samples obtained from cultures and from direct PCR on biopsies (127 in total). If subjects were positive by both techniques, only the biopsy amplified sample was included in this analysis. None of the young children had mixed cultures with relation to *cagA*, *vacAs *or *vacAm *alleles. Young children also exhibited lower levels of the toxigenic genes than any of the adult groups. This difference was only statistically significant (P≤0.02) when isolates obtained from children were compared with those from adults aged less than 60 years for *cagA *and *s1 *allele of *vacA*, and when compared with isolates from adults aged 41-59 years for *m1 *region of v*acA*. However, the sample size in children was small and therefore the difference between children and adults should be interpreted with caution. The prevalence of virulence genes was age-dependent. For *cagA*, *vacAs *and *vacAm *the virulent genotype was most common among the 30-40 year age group and less common in younger and older age groups. This association was statistically significant (p < 0.05) for *cagA *and v*acAs *and not for the mid region of *vacA *gene and *iceA *alleles (p>0.05, table 3). Only 1 elderly subject (70 years) was found to have mixed colonization with *vacAs1*/*s2*. The situation with *iceA *was more complicated, with a large number of individuals exhibiting mixed *iceA1*/*iceA2 *colonization.

**Table 3:** Variation in frequency of alleles with age from samples obtained by PCR directly from biopsies or subcultured *H. pylori*.

Age (years)	Total	*cagA*+	*cagA*-	*cagA*+&-	Not amplified	P-value
1.5-2.5	6	1(16.7)	5(83.3)	0(0)	0(0)	

9-29	54	35(64.8)	10(18.5)	8(14.8)	1(1.8)	

30-40	31	22(71.0)	2(6.5)	6(19.3)	1(3.2)	

41-59	24	15(62.5)	4(16.7)	4(16.7)	1(4.2)	

>=60	12	2(16.7)	5(41.7)	5(41.7)	0(0)	0.001

						

**Age (years)**	**Total**	***s1***	***s2***	***s1*&*s2***	**Not amplified**	**P-value**

1.5-2.5	6	1(16.7)	5(83.3)	0(0)	0(0)	

9-29	54	43(79.6)	9(16.7)	0(0)	2(3.7)	

30-40	31	27(87.1)	3(9.7)	0(0)	1(3.2)	

41-59	24	18(75.0)	5(20.8)	0(0)	1(4.2)	

>=60	12	5(41.7)	6(50.0)	1(8.3)	0(0)	<0.001

						

**Age (years)**	**Total**	***m1***	***m2***	***m1*&*m2***	**Not amplified**	**P-value**

1.5-2.5	6	1(16.7)	5(83.3)	0(0)	0(0)	

9-29	54	25(46.3)	17(31.5)	10(18.5)	2(3.7)	

30-40	31	16(51.6)	8(25.8)	5(16.1)	2(6.5)	

41-59	24	12(50.0)	4(16.7)	4(16.7)	4(16.7)	

>=60	12	2(16.7)	7(58.3)	3(25.0)	0(0)	0.103

						

**Age (years)**	**Total**	***iceA1***	***iceA2***	***iceA1*&*2***	**Not amplified**	**P-value**

1.5-2.5	6	3(50.0)	1(16.7)	2(33.3)	0(0)	

9-29	54	3(5.6)	19(35.2)	29(53.7)	3(5.6)	

30-40	31	6(19.3)	7(22.6)	15(48.4)	3(9.7)	

41-59	24	4(16.7)	10(41.7)	7(29.2)	3(12.5)	

>=60	12	2(16.7)	6(50.0)	4(33.3)	0(0)	0.065

## Discussion

In this study, we describe the comparison between results obtained from direct PCR to detect *H. pylori *from gastric biopsies in West Africa, compared to PCR of bacterial isolates obtained from the same set of gastric biopsies. Both techniques produced different success rates, as set out in table 1 and both failed to detect *H. pylori *in a significant proportion of infections. We agree with Park et al [14] in that direct PCR can produce inconsistent results, and tend to underestimate the prevalence of specific virulence factors (table 2). However, in this study, we detected a good consistency of genotypes between both techniques consistent with what was reported in a similar study [10].

Our data differs in that we experienced considerably greater difficulty in obtaining pure subcultures of *H. pylori *from gastric biopsies than Park, with a consequently higher failure rate. We have been involved in studies cultivating *H. pylori *from gastric biopsies from populations throughout the world, and it is our personal observation that sub-culture failure is a particular problem amongst West African isolates, as encountered in the present study. The reasons for this are not immediately apparent.

As a consequence of this problem, not all biopsies from which virulence factor DNA was amplified yielded a primary isolation of *H. pylori*, and there was a significant loss of isolates at subculture. PCR from subcultures gave higher rates of mixed colonization for *cagA *and *vacA *genes than direct PCR of biopsies, in contrast to the situation reported elsewhere with higher culture success rates [14]. This may have been due to artifact, either by enhancement of a minor strain from within the stomach, or due to modification of genome during culture [15]. In our hands, therefore, direct PCR produced more positive results, gave rise to fewer concerns about the development of artifact, and was more rapid and convenient.

Our data also indicate that there may be PCR inhibitors or potent nucleases in some gastric biopsies. This is consistent with findings in similar studies [12,14]. Their occasional presence and the underestimated prevalence of specific virulence factors by direct PCR illustrate that culture can be a useful complement to direct PCR for studies in which complete ascertainment of *H. pylori *virulence factor genotypes, including mixed colonization, is desired.

We observed a difference in predominant genotype with subjects' age. Young children produced isolates that were more likely to be *cagA*-ve, and *VacAs2m2*, in contrast to adults who were more likely to harbor *cagA*+ve *VacAs1m1 *isolates. Children were also less likely to have mixed populations of *H. pylori *strains, which may relate to children aged 18 to 31 months being relatively recently colonized by *H. pylori*, compared to older individuals. The strains of *H. pylori *discovered in adult stomachs, at ages when typical *H. pylori *associated diseases develop, may be genotypically distinct from the original strains that first colonized young Gambian children. This could be due to recombination of the *H. pylori *genome over the course of decades [16,17] and/or re-exposure to novel strains, with more pathogenic strains circulating predominantly amongst adults.

## Conclusion

In order to detect the range of bacterial genotypes harbored by individual patients, direct PCR proved slightly superior to isolation of *H. pylori *by biopsy culture in our hands, but the techniques were complementary to each other, and the use of both together produced the most complete picture. Despite the lower success rate and greater cost of *H. pylori *culture relative to PCR directly from biopsies, culturing *H. pylori *is still important for antibiotic susceptibility tests that could guide therapy and other phenotypic tests such as bacterial adherence, *cagA *and *vacA *action on mammalian cells, expression of other colonization and virulence traits for which PCR alone is unsuitable.

## Methods

### Patients

Ethical approval of study protocols was obtained from the joint Gambian Government MRC Ethical Committee and from The London School of Tropical Medicine and Hygiene.

169 gastric antral biopsies were obtained from adult subjects (50 female and 71 male) undergoing routine diagnostic endoscopy after obtaining informed consent. These subjects were consecutive patients attending the MRC endoscopy clinic for whom the endoscopist decided it was appropriate to take biopsies for research as well as clinical purposes. Patients with severe oesophago-gastroduodenal disease, including gastro-oesophageal varices or gastric cancer, were therefore not included in the study.

In addition, gastric biopsies were obtained, after informed parental consent, from 21 children aged 18 to 31 months, who were undergoing endoscopic small bowel biopsy because of suspected enteropathy.

The biopsies were immediately stored in Brain Heart Infusion (BHI) broth containing 20% glycerol and transported in ice to the laboratory for processing or stored at -70°C until used.

### Culture

Endoscopic biopsies were spread on the surface of selective Columbia-blood agar (Unipath, Basingstoke, UK) supplemented with 10% sheep blood (TCS Biosciences, UK), 2% vitox (Unipath, Basingstoke, UK) and the following antibiotics: trimethoprim (5 μg/ml), vancomycin (6 μg/ml), polymixin B (10 μg/ml), bacitracin (200 μg/ml), nalidixic acid (10 μg/ml), and an antifungal amphotericin B (8 μg/ml) [18]. The inoculated plates were incubated in a micro-aerobic atmosphere at 37°C for 5-7 days. Isolation and identification of *H. pylori *was made by colony morphology, Gram stain, oxidase, urease and catalase activity. Strains were preserved in BHI broth containing 20% glycerol and stored at -70°C.

### DNA extraction from cultures

DNA was prepared by harvesting a confluent growth of pooled *H. pylori *population from agar media and extracted using a commercial kit (QiagenR DNA Mini Kit, UK) as per manufacturer's guidelines. The DNA was stored at -20°C until used for gene amplification.

### DNA extraction directly from biopsies

Total genomic DNA was extracted from the biopsy samples by using a combination of the QIAamp DNA isolation kit (Qiagen, UK) and a bead-beater method. Briefly, biopsies were lysed in 180 μl of QIAamp ATL buffer and 20 μl of proteinase K for 1 h at 56°C. Glass beads of different diameters (0.1 mm, 0.5 mm and 1 mm, Sigma) were added, and samples were homogenized in a FastPrep FP120 bead-beater (Bio101, Savant Instruments) for 30 sec at 4 m/s and incubated for an additional hour at 56°C. 200 μl of AL buffer were added to the lysate and samples were incubated for 30 min at 70°C. After the addition of 200 μl absolute ethanol, lysates were purified over a QIAamp column as specified by the manufacturer.

### PCR amplification of *H. pylori *16s rRNA

PCR was performed on extracted DNA from biopsies and also from cultures using *H. pylori 16s *rRNA specific PCR ["*Hp16s*"] as previously described [19] under the following conditions: 35 cycles of 95°C for 30s, 60°C for 30s and 72°C for 30s and an extension time of 72°C for 5 min. The amplified genes were detected by electrophoresis in a 1.5% agarose gel with ethidium bromide (500 ng/ml) and bands visualized using Gel Doc 2000 (Bio-Rad laboratories, Milan, Italy).

### PCR to detect genotypes

PCR was performed to detect *cagA*, *vacA *genes, *iceA1 and iceA2 *using previously described methods [20], under the following general conditions: 30 cycles of 94°C for 1 min, 55°C for 1 min and 72°C for 1 min. The amplified genes were detected by electrophoresis in a 1.5% agarose gel with ethidium bromide and bands were visualized using Gel Doc 2000 (Bio-Rad laboratories, Milan, Italy). The primers used are listed in table 4.

**Table 4:** Primers used in this study

Region	Primer	Nucleotide sequence	bp	reference
*H. pylori 16sRNA*	Hp1	ctg gag aga cta agc cct cc	109	[19]
	Hp2	att act gac gct gat tgt gc		

*cagA*	cagA-F	gat aac agg caa gct ttt gag g	349	[20]
	cagA-R	ctg caa aag att gtt tgg cag a		

*cag empty site*	Luni-1	aca ttt tgg cta aat aaa cgc tg	535	[20]
	R5280	ggt tgc acg cat ttt ccc tta atc		

*vacA s1 & vacA s2*	Va1-F	atg gaa ata caa caa aça cac	s1 259	[20]
	Va1-R	ctg ctt gaa tgc gcc aaa c	s2 289	

*vacA m1a*	Va3-F	ggt caa aat gcg gtc atg g	290	[20]
	Va3-R	cca ttg gta cct gta gaa ac		

*vacA m2*	Va4-F	gga gcc cca gga aac att g	352	[20]
	Va4-R	cat aac tag cgc ctt gca c		

*iceA1*	1048-F	gct tgt aac gat aag aaa cgc cag at	297	[20]
	1345R	gga atg agc ttg tat tta gag ccg at		

*iceA2*	ICEA2-F	gtt ggg tat atc aca att tat	229	[20]
	ICEA2-R	ttr ccc tat ttt cta gta ggt	334	

### Statistics

Percentage agreement was calculated to compare *H. pylori *genotypes obtained by PCR performed directly on gastric biopsies with the genotypes obtained by PCR of DNA extracted from bacteria cultured. In addition, we report the kappa statistic which allows for chance agreement (kappa = 0 corresponds to no agreement beyond that expected by chance and kappa = 1 represents perfect agreement).

We studied the prevalence of genotypes within different age categories. The null hypothesis of no association between prevalence and age was tested using Fisher's exact test.

## Competing interests

The authors declare that they have no competing interests and the funders had no role in study design, data collection and analysis, decision to publish, or preparation of the manuscript.

## Authors' contributions

JET, RAA and DEB conceived the study. OS performed all the experiments, analysis and wrote the paper with JET, DEB and RAA. TC, JET, MT, RW collected all biopsies from subjects referred for clinical diagnoses. VT participated in consenting of patients and preparing the patients for endoscopy. CB was involved in the statistical analysis. All authors read and approved the final manuscript.
